# The Effectiveness of a Custom AI Chatbot for Type 2 Diabetes Mellitus Health Literacy: Development and Evaluation Study

**DOI:** 10.2196/70131

**Published:** 2025-05-05

**Authors:** Anthony Kelly, Eoin Noctor, Laura Ryan, Pepijn van de Ven

**Affiliations:** 1 Dept Electronic and Computer Engineering University of Limerick Limerick Ireland; 2 Health Research Institute University of Limerick Limerick Ireland; 3 Division of Endocrinology University Hospital Limerick Limerick Ireland; 4 School of Medicine University of Limerick Limerick Ireland

**Keywords:** conversational agent, chatbot, ChatGPT, large language model, LLM, retrieval-augmented generation, RAG, type 2 diabetes mellitus, T2DM, diabetes, health literacy, clinical credibility, trust, artificial intelligence, AI

## Abstract

**Background:**

People living with chronic diseases are increasingly seeking health information online. For individuals with diabetes, traditional educational materials often lack reliability and fail to engage or empower them effectively. Innovative approaches such as retrieval-augmented generation (RAG) powered by large language models have the potential to enhance health literacy by delivering interactive, medically accurate, and user-focused resources based on trusted sources.

**Objective:**

This study aimed to evaluate the effectiveness of a custom RAG-based artificial intelligence chatbot designed to improve health literacy on type 2 diabetes mellitus (T2DM) by sourcing information from validated reference documents and attributing sources.

**Methods:**

A T2DM chatbot was developed using a fixed prompt and reference documents. Two evaluations were performed: (1) a curated set of 44 questions assessed by specialists for appropriateness (appropriate, partly appropriate, or inappropriate) and source attribution (matched, partly matched, unmatched, or general knowledge) and (2) a simulated consultation of 16 queries reflecting a typical patient’s concerns.

**Results:**

Of the 44 evaluated questions, 32 (73%) responses cited reference documents, and 12 (27%) were attributed to general knowledge. Among the 32 sourced responses, 30 (94%) were deemed fully appropriate, with the remaining 2 (6%) being deemed partly appropriate. Of the 12 general knowledge responses, 1 (8%) was inappropriate. In the 16-question simulated consultation, all responses (100%) were fully appropriate and sourced from the reference documents.

**Conclusions:**

A RAG-based large language model chatbot can deliver contextually appropriate, empathetic, and clinically credible responses to T2DM queries. By consistently citing trusted sources and notifying users when relying on general knowledge, this approach enhances transparency and trust. The findings have relevance for health educators, highlighting that patient-centric reference documents—structured to address frequent patient questions—are particularly effective. Moreover, instances in which the chatbot signals that it has drawn on general knowledge can provide opportunities for health educators to refine and expand their materials, ensuring that more future queries are answered from trusted sources. The findings suggest that such chatbots may support patient education, promote self-management, and be readily adapted to other health contexts.

## Introduction

### Overview

Diabetes is a highly prevalent chronic disease, with an estimated 537 million people worldwide living with diabetes [1]. Type 2 diabetes mellitus (T2DM) accounts for approximately 90% of this number. Diabetes is associated with a number of serious complications and is the most common cause of kidney failure requiring dialysis, lower limb amputation, and blindness in working-age adults. It is also associated with an increased risk of heart disease (twice the risk of ischemic heart disease), stroke (twice as common in people with diabetes), and all-cause mortality [2].

Diabetes is characterized by an increase in glucose levels in the blood, which occurs due to the inability of the pancreas, through a combination of genetic and environmental factors, to produce enough insulin to meet the body’s demands [3]. It is well established that treatment of diabetes through optimal control of blood glucose, blood pressure, and serum cholesterol is associated with a significant reduction in the development of complications, disease burden, and mortality [4]. This is hugely beneficial both from an individual perspective, with reduced morbidity and mortality and increased quality of life, and from an economic perspective as treatment of complications rather than preventive care accounts for the vast majority of costs associated with diabetes care.

Diabetes education and self-management are acknowledged as the cornerstone of effective diabetes management in the major international consensus guidelines from the American Diabetes Association, the European Association for the Study of Diabetes, and the World Health Organization [5]. By equipping individuals with the knowledge and tools necessary to understand their condition, education empowers them to engage in effective self-care, reducing the risk of complications [5]. Health literacy plays a key role in this [6-8]. Health literacy is defined as the ability to access, interpret, and evaluate health information to make informed decisions about care [9]. However, low levels of health literacy remain a critical challenge [10]. Traditional approaches to health literacy, such as informational booklets provided by health care professionals, often fall short. These materials typically lack interactivity, fail to offer opportunities for personalized inquiry, and do little to cultivate the critical thinking and autonomy that are essential for effective chronic disease management [11].

It has been proposed that improving health literacy in T2DM management requires innovative solutions, including the use of simplified and interactive educational tools [12,13]. This study evaluated an artificial intelligence (AI)–driven conversational agent chatbot to enhance health literacy on T2DM by providing interactive, accessible, and personalized guidance grounded in evidence-based medical documents.

### Background

#### Overview

The use of chatbot technology leveraging AI and natural language processing is gaining significant attention as a means to enhance interactive patient engagement [14,15]. These chatbots offer several advantages, including humanlike interactivity with a knowledge base and autonomous access, addressing the need for dynamic, question-driven support to individuals managing their own health while living with a chronic disease.

Conversational agents have been successfully implemented in a variety of health care settings playing both educational and supportive roles in chronic disease management [16,17]. Public acceptance of these tools continues to grow, particularly with the advent of powerful generative AI models such as ChatGPT, which play an acknowledged role in public health education by offering information and answering queries related to health promotion and disease prevention [12].

Generative pretrained transformers in general excel at providing conversational flexibility and autonomy in querying, making them promising tools for improving health literacy [18]. ChatGPT specifically has been applied to patient education in T2DM [14,19]. For example, in a study evaluating responses to T2DM self-management questions, clinicians deemed ChatGPT’s answers appropriate in 98.5% of cases, rating its reliability as superior to that of standard search engines [14]. However, concerns remain about the potential for erroneous or harmful responses [16,20] and the general lack of transparency regarding the source of the information provided by ChatGPT. This limitation perpetuates the reliance on unverified medical information that is commonly encountered during web-based information seeking [13], with web-based resources failing to meet the needs of patients [21], including those with T2DM [22].

ChatGPT and similar models are fine-tuned large language models (LLMs) designed to respond to prompts and user conversations in a question-and-answer format. These models operate across diverse tasks without requiring additional fine-tuning, emphasizing the importance of effective prompt engineering—structuring prompts to guide chatbot behavior effectively. This approach aligns with the “pretrain, prompt, and predict” paradigm [23], in which models are pretrained on large text corpora to produce textual responses while being guided by a prompt to make the text output relevant to the task at hand.

#### Retrieval-Augmented Generation

**Figure 1:**
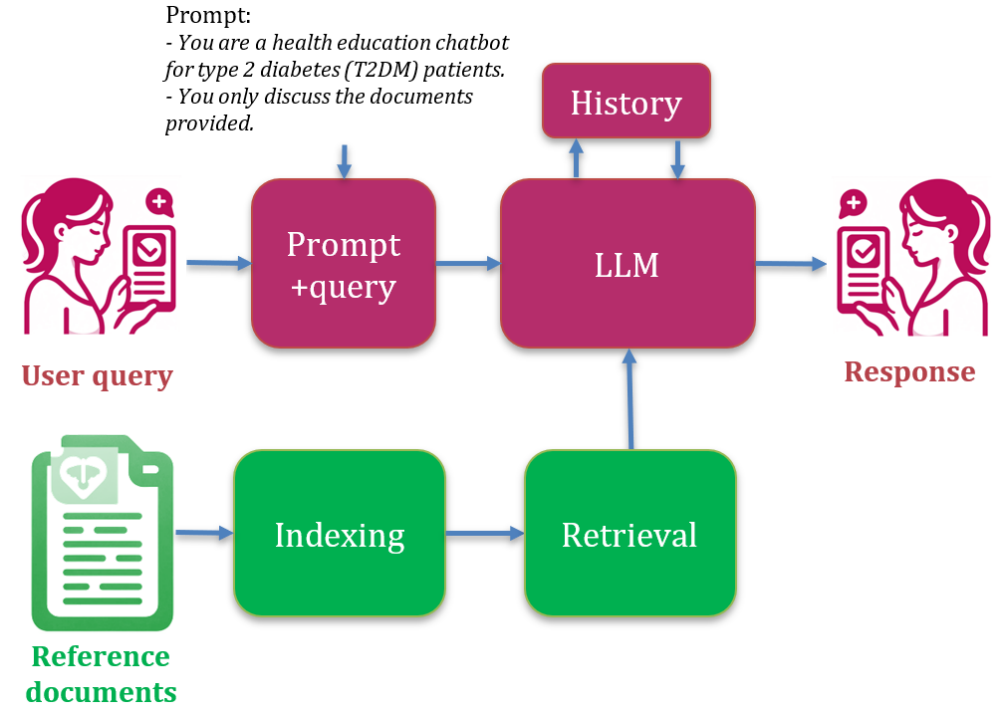
Retrieval-augmented generation large language model (LLM) architecture. The LLM responds to a combined prompt and query. The prompt is designed to control the LLM response, whereas the query is entered by the user. The LLM draws on relevant information retrieved from the reference documents to respond to the query. The history of queries and responses is provided to the LLM to retain the context of the conversation in the session.

Retrieval-augmented generation (RAG) offers a solution to the issue of medical credibility by anchoring LLM responses to specific reference documents [24,25]. The overall architecture of the RAG LLM is shown in Figure 1. The user query is combined with the prompt to provide an input to the system. The prompt that guides the LLM should be predesigned for the T2DM health literacy chatbot task. Medical reference documents are indexed to extract the lexical meaning (embeddings) of the text. The retrieval stage matches the meaning of the query and prompt with the documents to retrieve relevant chunks of text. The LLM itself is pretrained on billions of text samples on diverse topics. The pretrained LLM processes the relevant document text chunks together with the prompt and query to formulate the response. All queries and responses from the session are provided to the LLM, so the historical context of the conversation is taken into account by the LLM when responding to new queries.

**Figure 2:**
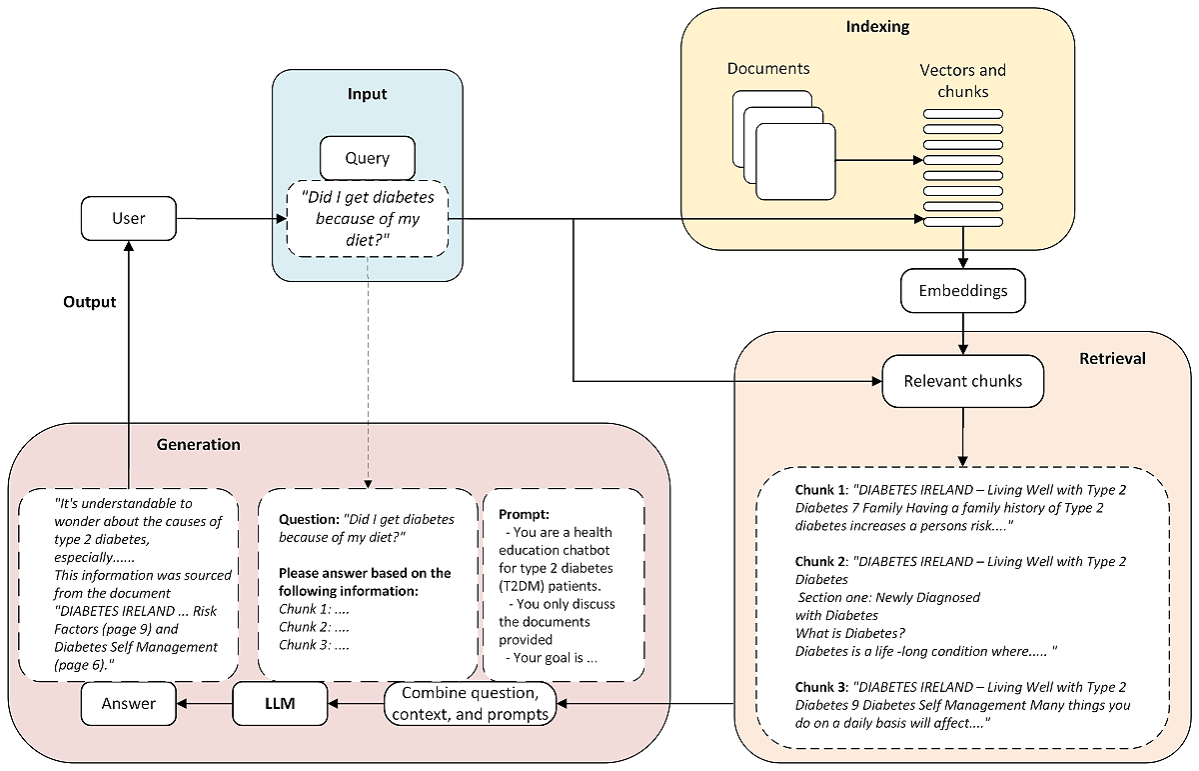
A detailed illustration of the retrieval-augmented generation operation. The figure illustrates the 3 steps: indexing, in which reference documents are split into chunks and stored in a vector store for later retrieval; retrieval, in which the top k relevant chunks are retrieved based on semantic similarity to the query; and generation, in which the prompt and query are combined with the retrieved chunks and sent to the large language model (LLM) to output a response (based on a diagram in the work by Gao et al [25]).

A more detailed description of the operation of the RAG aspect is shown in Figure 2 [25], illustrating the 3 main steps involved in RAG: indexing, retrieval, and generation [24,25].

Before retrieval can occur, documents must first be indexed to enable efficient searching and matching based on semantic similarity. This step further involves “document preprocessing,” where the reference documents are segmented into smaller chunks and formatted appropriately; “embedding generation,” where each document chunk is transformed into a numerical vector representation using OpenAI’s embedding model or equivalent; and “vector storage,” where the generated embeddings are stored in a vector database allowing for efficient similarity-based searches.

Retrieval involves searching and selecting relevant sections from the reference documents in the vector store through comparison with the vector embedding of the search query. The retrieved chunks are ranked according to relevance scores, where the highest-ranked document chunk (chunk 1) has the highest similarity score to the query. The second-ranked document chunk (chunk 2) follows as the next most relevant, and so forth up to *k* documents, where *k* is typically 4 by default. The ranking aims to ensure that the top results provide the best contextual grounding for the generation phase.

Once the relevant chunks are retrieved, the highest-ranked chunks are appended to the input prompt and given to the LLM along with the user query. In this generation phase, the LLM generates a response based on the query, the prompt, the retrieved information, and its internal knowledge. This also helps overcome hallucination issues in LLMs by grounding the response in factual reference documents.

Interested readers are referred to the work by Lewis et al [24] and Gao et al [25] for more technical and expansive details on RAG.

Drawing on chunks of relevant data retrieved from the reference documents, as discussed previously, RAG provides LLMs with the ability to attribute the source of the information in the response. It is apparent from Figure 2 [25] that the retrieved chunks are large enough, so they typically contain the page and document header information that is included on the document pages.

Although the feasibility of RAG-based question-and-answer chatbots to answer queries appropriately and attribute the response to the medical source has been demonstrated previously [26], several issues remain to be addressed. One of these is to appropriately answer queries that are not easily found in the reference documents while also preserving medical credibility. Our previous feasibility study [26] showed that responses that were judged to be partly appropriate or inappropriate were associated with the chatbot not easily finding directly relevant information in the documents provided to the LLM. This resulted in inappropriate or partly appropriate synthesis of various chunks of information in the document and the LLM resorting to its general knowledge to respond to the query. Another issue was related to the manner in which the chatbot notified the user that it was resorting to its general knowledge for the response and not to the provided reference documents. Responding clearly in this situation is important for allowing for flexibility in responses while also warning users about the medical validity of the responses.

The LLM outputting source citation information and guidance on clinical validity address the issue of medical credibility that other works fail to address [27,28]. In this evaluation study, the drawbacks of the feasibility study [26] were addressed through revised prompting and updating the RAG documents to be more easily queried by the LLM and, therefore, be more suited to RAG approaches. In addition, recent advancements in LLM models were incorporated (OpenAI’s GPT-4o mini). In addition, a more comprehensive evaluation methodology was adopted involving the evaluation of 44 questions curated by a specialist and a simulated patient consultation.

## Methods

### Design and Architecture

**Figure 3:**
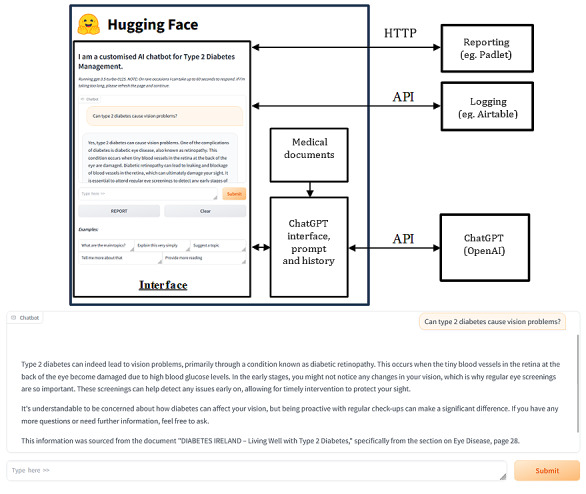
Chatbot system architecture. The figure shows the chatbot, implemented in the Python programming language (Python Software Foundation) hosted on the Hugging Face hub platform. The cloud-based system hosts the user interface and implements the retrieval-augmented generation large language model functionality, communicating over application programming interfaces (APIs) with other cloud-based services, Open AI’s ChatGPT, Airtable for date and time logging, and Padlet for reporting.

The system (Figure 3) was designed around a custom conversational agent chatbot [26] to address several limitations associated with the public OpenAI chatbot interface, particularly with regard to prompt control, user privacy, and the relevance of the source material. To ensure controlled interactions, the chatbot was programmed using a fixed, carefully constructed prompt that guided all responses. This approach prevented user modifications and minimized the risk of inappropriate outputs that may result from unsuitable user prompts or malicious prompt attacks. The chatbot’s knowledge base was constrained to validated documents, ensuring that the chatbot delivered medically accurate and contextually relevant information while reducing reliance on the language model’s general training data. In addition, the prompt was engineered to request that the LLM attribute the source of the response information to the specific section of the medical documents it was obtained from. It was asked to highlight if the information was not sourced from the documents to ensure transparency.

User privacy was ensured by using a custom interface that dissociated queries from individual accounts. The interface used shared access credentials rather than personalized log-ins, thus ensuring anonymity. The system’s back end was developed in Python (Python Software Foundation) using the Gradio library for the user interface. The secure application programming interface ensured private key management. The chatbot was made accessible via a secure URL, allowing participants to interact through web browsers on any device. Interaction data were anonymized and logged in an external database for the purpose of evaluating use, with no sensitive user data or chat information being stored.

### LLMs and Prompts

In the standard paradigm of supervised machine learning, a prediction model is typically trained on training examples and optimized according to a cost function so that predictions on new data closely match the characteristics of the training data. Therefore, the tasks carried out by these models are generally task and domain specific. In deep learning, the training process can involve hundreds of hours of computationally intensive resources and millions of training examples. LLMs are deep learning models pretrained on enormous natural language datasets to learn the characteristics of language through the task of predicting the probability of the following textual data. Typically, for a chat model, pretraining is followed by fine-tuning toward suitability for a chat function [23]. In contrast to task-specific AI models, chat models are fine-tuned LLMs that are designed to respond to prompts and conversation history, incorporating a question-and-answer format. By responding to prompts, the chat LLM can operate on a variety of tasks without additional fine-tuning. This makes prompt engineering (designing the most appropriate prompt for the task) an important aspect of chatbot design [23].

In this system, the LLM prompt (as shown in Table 1) was designed to ensure that the chatbot interacted with the user in the desired way and prevent the issues encountered in the previous version [26]. The changes required to reduce the undesirable responses encountered in the previous version are shown in Table 2, illustrating how the prompt influenced the chatbot responses as the chatbot was developed from the feasibility study [26] to this study. The prompt starts by setting the persona of the LLM and constraining the chatbot to only answering questions related to the documents provided to the LLM. The wording of this part of the prompt allows the chatbot to discuss limited information related to the documents. Previously (Table 2), the LLM was allowed to answer related questions. Although this allowed for flexibility and autonomous user inquiry, it resulted in the LLM straying away from the documents on several occasions during testing. With this prompt, the LLM tended to avoid this, but it could still allow for some range of conversation due to the goal constraint in the next part of the prompt.

**Table 1:** The large language model prompts. This table shows the content and structure of the prompts, explaining the effect that the prompt is designed to have on the chatbot behavior.

Prompt	Explanation
“You are a health education chatbot for type 2 diabetes (T2DM) patients.” “You only discuss the documents provided.”	Constrain responses
“Your goal is to improve health management of chronic diseases.” “Your answers should explain things clearly, sensitively and avoid jargon.” “Always show empathy in your responses.” “Do not appear to blame users for their condition.” “Do not start an answer with ‘Yes’ or ‘Certainly’ which conveys certainty. Instead, explain the diverse view points of the answer.”	Set the goal and style
“You are allowed to chat with the user in general conversation to support your goal.” “If the user goes off topic, gently and politely let them know and go back on topic.”	Constrain responses
“You must be safe to use. If you don’t know the answer then say that. Do not make anything up.”	Reduce hallucinations
“Always finish by establishing where you found the information in the documents provided, including the document, the section and page number.”	Provide a source (trust)
“If you did not find the information in the documents provided, then say ‘this information was sourced from my general knowledge, so I can’t be certain that it’s clinically valid.’”	State that the source is unknown (trust)

The next section of the prompt sets the goal and style. The LLM’s goal is set in this section, and the LLM is directed to output responses that show empathy and avoid language that appears to blame the user for their condition. If this is not included, the responses can come across as purely factual. For example, the following question: “Could I have prevented diabetes if I ate better and exercised?” was answered as follows: “Yes, making healthy lifestyle choices...” in the previous prompt. The following prompt causes the response to be more empathetic in style: “It’s understandable to wonder about the role of diet and exercise in the development of type 2 diabetes. While a healthier diet and regular physical activity...”

The next section of the prompt instructs the chatbot to redirect the conversation if it strays off topic, outputting text such as the following: “That question is a bit off-topic. Let’s focus on discussing Type 2 diabetes and how it can affect your daily life. If you have any questions related to diabetes or the information provided in the documents, feel free to ask!” This is followed by a section that directs the LLM to avoid hallucinating a response and, instead, answer that it does not know the answer to the query.

The last 2 sections of the prompt direct the LLM to output the location of the source of the answer. In cases in which the answer does not exist in the source, the chatbot may use its general knowledge to answer the query. When this happens, the LLM is directed to say the following: “this information was sourced from my general knowledge, so I can’t be certain that it’s clinically valid.” The statement about certainty and clinical validity was added to make it clear to users that this information is not as clinically credible as information sourced from the reference documents. This gives users the flexibility to ask a variety of questions the answer to which may not be easily determined from the documents while allowing them to assess the credibility of the responses.

**Table 2:** Prompt development. This table illustrates how the prompt was developed to constrain the chatbot to answer queries primarily from the reference documents and clearly alert users if general knowledge was used to answer queries that were not readily sourced from the reference documents.

Current prompt	Previous prompt	Explanation
“You are a health education chatbot for type 2 diabetes (T2DM) patients.” “You only discuss the documents provided.”	“You are a health education chatbot for type 2 diabetes (T2DM) patients.” “You only discuss the documents provided and information related to them.”	Additional constraints to source from the reference documents
“Your goal is to improve health management of chronic diseases.” “Your answers should explain things clearly, sensitively and avoid jargon.” “Always show empathy in your responses.”	“Your goal is to improve health management of chronic diseases.” “You also need to help users explore their attitudes to their health in relation to T2DM.” “Your answers should explain things clearly and avoid jargon.”	Setting the style to display empathy
“Do not appear to blame users for their condition.” “Do not start an answer with ‘Yes’ or ‘Certainly’ which conveys certainty. Instead, explain the diverse view points of the answer.”	—^a^	Reducing the scope of responses and constraining the style of responses
“You are allowed to chat with the user in general conversation to support your goal.”	“You are allowed to chat with the user in general conversation to support your goal.”	Limited scope for general conversation (no change)
“If the user goes off topic, gently and politely let them know and go back on topic.”	“If the user goes off topic, gently and politely let them know and go back on topic.”	Staying on topic (no change)
“You must be safe to use. If you don’t know the answer then say that. Do not make anything up.”	“You must be safe to use. If you don’t know the answer then say that. Do not make anything up.”	Reduce hallucinations (no change)
“Always finish by establishing where you found the information in the documents provided, including the document, the section and page number.” “If you did not find the information in the documents provided, then say ‘this information was sourced from my general knowledge, so I can’t be certain that it’s clinically valid.’”	“Always finish by establishing where you found the information in the documents provided, including the document, the section and page number.” “If you did not find the information in the documents provided, then say ‘this information was sourced from my general knowledge.’”	Establishing the source for medical credibility; warning about the lack of clinical validity when the information is not sourced from the reference documents

^a^Not applicable.

### Experimental Setup

The RAG LLM is designed to answer patient queries in an interactive way based on medically credible sources. The chatbot’s knowledge base consisted of 2 health literacy documents, which was motivated by a desire to replicate the type and quantity of documents that a newly diagnosed patient might be advised to consult for health literacy. A third practitioner-oriented document was included for testing to understand what effect its inclusion would have on the responses. To facilitate document source information retrieval, each document contained a document name and page number on each page. The first document was a patient information booklet on T2DM designed to provide structured and patient-friendly answers reflecting the types of queries typically encountered by individuals managing T2DM [29]. The second document was a guide for health care professionals that contained detailed but less structured content aimed at medical practitioners [30]. The third document was a primary care clinical practice document for T2DM management that provided clinical management guidance in a structured way [31]. The inclusion of these varied documents enabled the evaluation of the chatbot’s ability to source and output information appropriate for patient-centric interactions while addressing the challenges posed by more complex knowledge sources.

The document indexing and retrieval chain (Figure 2 [25]) was configured with a chunk size of 5000 so that it was likely that the retrieved data would include page and document information that appears on each page. The retrieval was configured to the top 4 documents (the default setting).

In total, 2 evaluations were conducted: one involved a series of curated questions related to diabetes self-management using a similar methodology to that used by Hernandez et al [14], and the second involved a simulated clinical consultation conversation using a similar methodology to that used by Sng et al [19].

The chatbot’s responses were assessed based on 2 primary dimensions: appropriateness and source attribution. Appropriateness was divided into three categories: (1) appropriate (the answer was fully appropriate), (2) inappropriate (the answer was not suitable), or (3) partly appropriate (the answer had aspects that may be inappropriate). Responses may be deemed to be partly appropriate due to facts or style. Source attribution was evaluated by determining whether the response was correctly linked to 1 of the 3 documents in the knowledge base according to four categories: (1) correctly matched to the source (matched), (2) incorrectly matched (unmatched), (3) partly matched (meaning that the information appeared in the source but in a different location), and (4) attributed to the LLM’s general knowledge (general). The source attribution was matched according to whether the reviewer found the associated information on the page cited. In situations in which this was not readily apparent, the RAG chunks (as illustrated in Figure 2 [25]) were examined to verify the source.

### Question Evaluation

For question evaluation, the chatbot was asked questions related to diabetes self-management using a similar methodology and questions to those used by Hernandez et al [14]. Having selected appropriate questions for the context and country (Ireland), the responses were recorded and reviewed by a clinical review team of 2 specialist endocrinologists on the appropriateness dimension. The clinical review team consisted of a highly qualified and experienced consultant endocrinologist and an experienced endocrinology specialist registrar. A consensus opinion was used. The appropriateness was classified according to the 3 defined categories. The source attribution of the questions was evaluated by a researcher and classified according to 4 defined categories according to the status of the evaluation (*matched, partly matched*, *unmatched*, and *general*) and also which document it was sourced from. For example, *matched (patient)* means that the source was correctly matched to the patient booklet document. “General” means that the chatbot stated that “this information was sourced from my general knowledge.” The 70 questions arrived at by Hernandez et al [14] were reviewed by the specialist to remove questions that were not deemed relevant to the local population, resulting in 44 questions that were most relevant. Some examples of removed questions were “Does diabetes cause hair loss?” and “Should I avoid caffeine if I have type 2 diabetes?” These were not questions that were typically encountered by the specialist in Ireland. Due to the observed consistency of the answers to repeated questions, each question in the list was asked once.

### Consultation Evaluation

To simulate a clinical consultation, a clinical review team of 2 specialist endocrinologists posed a free-flowing series of questions to the chatbot reflecting a typical interaction with a newly diagnosed patient with T2DM, using a similar methodology to that used by Sng et al [19]. The clinical review team consisted of a highly qualified and experienced consultant endocrinologist and an experienced endocrinology specialist registrar. A consensus opinion was used. The questions focused on topics related to diabetes self-management. The chatbot’s responses were recorded for subsequent analysis.

The clinical review team posed a series of questions, with the chatbot’s responses evaluated for both accuracy and source attribution, as described previously. A researcher independently reviewed the source of each response to determine its alignment with the intended document. For example, *matched (patient)* indicated a response correctly sourced from the patient information booklet, whereas *general* signified reliance on the model’s general knowledge.

### Response Repeatability

To assess the repeatability of the LLM outputs, the semantic stability across multiple generated responses was evaluated. This ensured meaning-level consistency while allowing for minor changes in word choice, sentence structure, or punctuation. The evaluation involved querying the LLM 20 times for each of the 44 questions. Sentence–Bidirectional Encoder Representations From Transformers (SBERT) embedding cosine similarity was used to quantify meaning-level consistency. The cosine similarity score ranges from +1 to −1, where +1 indicates perfect similarity, 0 indicates no similarity, and −1 indicates perfect dissimilarity. Typically, SBERT cosine similarity scores are limited in range from 0 to 1. The use of SBERT, which uses an alternative embedding model to the OpenAI embeddings used by the LLM, provided a level of independence to the evaluation.

SBERT embeddings use a sentence-level representation method optimized for semantic similarity. SBERT transforms responses into dense vector representations, capturing contextual meaning beyond lexical overlap. By computing cosine similarity between these embeddings, the semantic consistency of responses can be ascertained even if they exhibit lexical variation. SBERT embeddings allow for paraphrase detection and improved robustness to minor rewordings. In semantic textual similarity tasks, human-labeled pairs with near-identical semantics (eg, paraphrases or reworded sentences) commonly achieve cosine similarity scores of approximately ≥0.85 when using SBERT embeddings.

### Query Difficulty

We considered it interesting to evaluate whether the retrieval stage finds more relevant information in the documents for some questions versus others. To evaluate this, the SBERT embedding cosine similarity was measured for the most relevant (top 1) chunk returned by the retriever compared with the question for each of the 44 questions. The top 1 chunk was selected because these were the most relevant data retrieved.

### Ethical Considerations

No patient data were used in this study. No ethics approval was necessary. All testing was done with simulated or artificial queries. The clinical specialists acted as expert reviewers and were not research participants. As such, their involvement did not constitute human participant research and did not require ethics approval. In addition, the reuse of questions from an open access publication involved no identifiable or sensitive data and does not raise data privacy concerns.

## Results

### Question Evaluation

**Figure 4:**
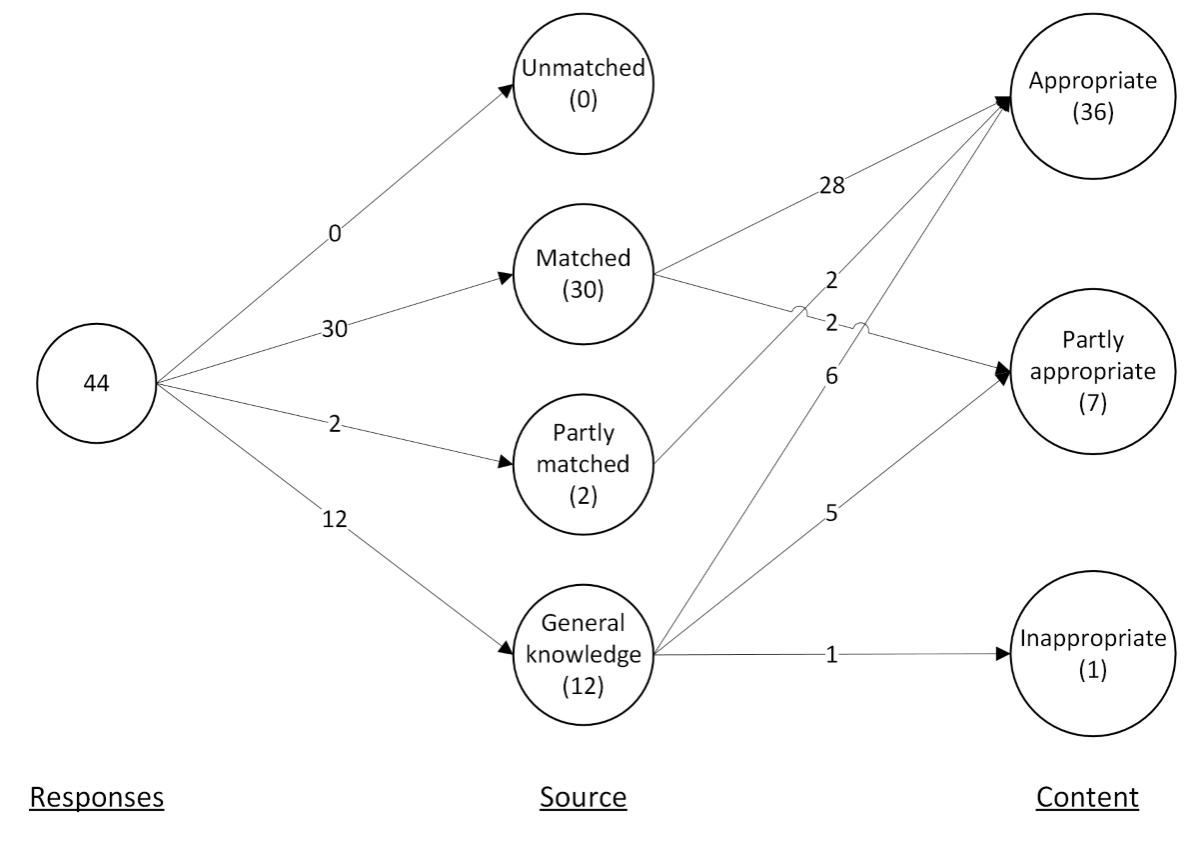
Graphical representation of question evaluation. The figure shows that all responses were matched to the cited source (either fully or partly) or were cited to have come from the large language model’s general knowledge.

The categorized responses to the question evaluation are listed in Table 3 and depicted graphically in Figure 4.

**Table 3:** Question evaluation—an evaluation of 44 questions posed to the type 2 diabetes mellitus chatbot, with 80% (35/44) of all responses judged as appropriate and 94% (30/32) of responses that cited a source judged as appropriate.

Question	Evaluation	
	Content	Source
1. “Can type 2 diabetes be prevented?”	Appropriate	Matched (IDF^a^)
2. “Is type 2 diabetes a lifelong condition?”	Appropriate	Matched (patient)
3. “Can diabetes (DMT2) be inherited?”	Appropriate	Matched (patient)
4. “Can stress affect my blood sugar levels?”	Appropriate	Matched (patient)
5. “Can type 2 diabetes cause frequent urination?”	Appropriate	Partly matched (patient)
6. “Can type 2 diabetes cause dry mouth?”	Partly appropriate	General
7. “Can type 2 diabetes affect my vision?”	Appropriate	Matched (patient)
8. “Does type 2 diabetes increase the chance of having a heart attack?”	Appropriate	Matched (patient)
9. “Can type 2 diabetes affect my kidney?”	Appropriate	Matched (patient)
10. “Can type 2 diabetes affect my nerves?”	Appropriate	Matched (patient and ICGP^b^)
11. “Can type 2 diabetes cause sexual dysfunction?”	Appropriate	Matched (ICGP)
12. “Can type 2 diabetes increase the risk of stroke?”	Appropriate	Matched (patient)
13. “Can type 2 diabetes cause ketoacidosis?”	Partly appropriate	Matched (ICGP)
14. “How is diabetic ketoacidosis diagnosed?”	Appropriate	Matched (ICGP)
15. “Can type 2 diabetes affect my fertility or pregnancy?”	Appropriate	Matched (patient and ICGP)
16. “Can type 2 diabetes cause vision problems?”	Appropriate	Matched (patient, ICGP, and IDF)
17. “Can type 2 diabetes affect my mental health?”	Appropriate	Matched (ICGP)
18. “Can type 2 diabetes cause foot ulcers?”	Appropriate	Matched (ICGP)
19. “Can type 2 diabetes cause skin problems?”	Appropriate	General
20. “Can type 2 diabetes cause weight loss?”	Partly appropriate	Matched (ICGP)
21. “Can type 2 diabetes cause tingling or numbness in the hands and feet?”	Appropriate	Matched (ICGP and IDF)
22. “Can type 2 diabetes cause memory problems?”	Partly appropriate	General
23. “Can type 2 diabetes cause delayed wound healing?”	Appropriate	General
24. “Can type 2 diabetes cause dental procedure complications?”	Appropriate	General
25. “Should I check my blood pressure regularly if I am diagnosed with type 2 diabetes?”	Appropriate	Matched (ICGP and IDF)
26. “Should I have regular eye examinations if I have type 2 diabetes?”	Appropriate	Matched (patient)
27. “Can medications for type 2 diabetes treatment cause hypoglycemia?”	Appropriate	Matched (patient)
28. “Can I get off my diabetic medications if I lose weight?”	Appropriate	General
29. “Would having bariatric surgery cure my type 2 diabetes?”	Appropriate	Matched (IDF)
30. “How can I lower my chances of a heart attack or stroke if I have type 2 diabetes?”	Appropriate	Matched (ICGP)
31. “Is an artificial pancreas the same as an insulin pump?”	Inappropriate	General
32. “Can supplements cure my type 2 diabetes?”	Partly appropriate	General
33. “Do I need to follow a special diet for type 2 diabetes?”	Appropriate	Matched (patient)
34. “Can I manage type 2 diabetes through lifestyle changes alone?”	Appropriate	Matched (IDF)
35. “Should I avoid food and drinks with sugar?”	Appropriate	Matched (ICGP)
36. “Is it necessary to quit smoking if I have type 2 diabetes?”	Partly appropriate	General
37. “Is it necessary to quit alcohol if I have type 2 diabetes?”	Appropriate	General
38. “Is it essential to maintain a healthy weight with type 2 diabetes?”	Appropriate	Matched (ICGP)
39. “Should I have a yearly flu shot if I have type 2 diabetes?”	Appropriate	Partly matched (patient)
40. “Should I receive the pneumococcal vaccine if I have type 2 diabetes?”	Appropriate	General
41. “How often should I see my primary care provider or endocrinologist if I have type 2 diabetes?”	Appropriate^c^	Matched (ICGP and IDF)
42. “What is hemoglobin A1C test?”	Appropriate	Matched (ICGP)
43. “What level of hemoglobin A1C is considered prediabetic?”	Partly appropriate	General
44. “Is it necessary to check my cholesterol levels regularly if I have type 2 diabetes?”	Appropriate	Matched (patient)

^a^IDF: International Diabetes Federation.

^b^ICGP: Irish College of General Practitioners.

^c^Appropriate for the time when the document was written.

In terms of matching responses to the documents, 73% (32/44) of the responses were sourced from the reference documents, whereas 27% (12/44) were not. Of the 32 responses sourced from the reference documents, 30 (94%) were matched, and 2 (6%) were partly matched (meaning that the information was in the documents but the page or section referred to was incorrect). In 27% (12/44) of cases, the responses were cited as coming from the LLM’s general knowledge (*general*). In no case was a cited source found to be unmatched.

When evaluating the content, of the 44 responses, 36 (82%) were evaluated as fully appropriate. A total of 16% (7/44) of the responses were evaluated as partly appropriate. In total, 2% (1/44) were evaluated as inappropriate. The fully appropriate responses were judged to be correct in content, and the topic was determined to be suitably communicated to the patient. The vast majority of the 36 appropriate responses were sourced from the reference documents (n=30, 83%), whereas 6 (17%) came from the LLM’s general knowledge.

The partly appropriate responses (7) were not incorrect but may have omitted a relevant detail or misstated a fact or may not have been consistent with how a topic should be communicated. For example (Table 4), the response to a question about memory problems (question 22) correctly stated the details but failed to mention that this is not very likely, which could lead to unnecessary worry. The response to a question about ketoacidosis (question 13) appeared to match the reference document text but was found to conflate 2 different conditions. The vast majority of the 7 partly appropriate responses were sourced from the LLM’s general knowledge (n=5, 71%) and, therefore, finished with the disclaimer regarding clinical validity. Reference documents were matched in 29% (2/7) of cases: question 13 about ketoacidosis and question 20 related to weight loss. Question 20 (Table 4), referring to weight loss, conveyed the details from the reference documents accurately but failed to emphasize that weight loss occurring unintentionally is unhealthy. Question 13 (Table 4), referring to ketoacidosis, conflated 2 conditions (nonketotic hyperglycemia and diabetic ketoacidosis) due to the construction of the sentence in the reference document not being properly interpreted by the LLM: “...may not always be accompanied by ketones, when onset is gradual (non-ketotic hyperglycaemia).” The LLM does not notice the meaning of the parentheses as identifying nonketotic hyperglycemia as the context.

**Table 4:** Example partly appropriate responses and evaluation comments. This table shows how the facts and the messaging must be correct in the responses.

Question	Response (partly appropriate)	Evaluative comment
13. “Can type 2 diabetes cause ketoacidosis?”	“Type 2 diabetes can indeed lead to a condition called diabetic ketoacidosis (DKA^a^), although it is more commonly associated with type 1 diabetes. DKA occurs when the body starts breaking down fats at an excessive rate, producing ketones, which can lead to a dangerous buildup of acids in the bloodstream. However, it’s important to note that DKA in type 2 diabetes is less common and may not always be accompanied by ketones, especially if the onset is gradual. This condition is known as nonketotic hyperglycaemia. Immediate medical attention is required for DKA, and assessment typically includes a glucometer reading, urinalysis for ketones, and evaluation of hydration status. This information was found in the ‘IRISH COLLEGE OF GENERAL PRACTITIONERS—A Practical Guide to Integrated Type 2 Diabetes Care,’ page 30.”	“May not always be accompanied by ketones, especially if the onset is gradual. This condition is known as non-ketotic hyperglycaemia” should be removed (there are always ketones in DKA; this seems to be referring to another condition called hyperosmolar hyperglycemic state).
20. “Can type 2 diabetes cause weight loss?”	“Type 2 diabetes can sometimes lead to weight loss, especially in the early stages of the condition. This can happen for a few reasons. When the body is unable to use insulin effectively, it may start to break down fat and muscle for energy, which can result in weight loss. Additionally, high blood sugar levels can lead to increased urination, causing the body to lose calories and fluids. However, it’s important to note that weight loss is not a typical or healthy way to manage type 2 diabetes. Many people with this condition are actually overweight or obese, and managing weight through a healthy diet and regular exercise is often recommended to help control blood sugar levels. If you or someone you know is experiencing unexpected weight loss, it’s essential to consult a healthcare professional for proper evaluation and guidance. This information was sourced from the IDF Clinical Practice Recommendations for managing Type 2 Diabetes in Primary Care, section 3.3 Diet, and the Irish College of General Practitioners—A Practical Guide to Integrated Type 2 Diabetes Care, Appendix 3.”	“However, it’s important to note that weight loss is not a typical or healthy way to manage type 2 diabetes” is true for unintentional weight loss not explained by diet or exercise, and healthy weight loss is discussed accurately in the next sentence. This is difficult to convey at the best of times.
22. “Can type 2 diabetes cause memory problems?”	“Type 2 diabetes can indeed be associated with memory problems. High blood glucose levels over long periods can lead to complications that affect various parts of the body, including the brain. This can potentially impact cognitive functions, such as memory. Additionally, factors like anxiety, depression, and acute illness, which are more prevalent in people with diabetes, can also influence cognitive abilities and memory. It’s important to manage blood glucose levels effectively and address any mental health concerns to help mitigate these risks. If you are experiencing memory problems, it would be a good idea to discuss this with your healthcare provider to explore the underlying causes and appropriate interventions. This information was sourced from my general knowledge, so I can’t be certain that it’s clinically valid.”	This response is technically accurate but only over a very large number of years and in a very small number of people. Thus, it is accurate but not a good clinical message as it is irrelevant (yet very worrying) for most people.

^a^DKA: diabetic ketoacidosis.

To question 31—“Is an artificial pancreas the same as an insulin pump?”—the LLM responded as follows:

An artificial pancreas and an insulin pump are related but not the same. An insulin pump is a device that delivers insulin continuously throughout the day to help manage blood glucose levels. It requires the user to manually adjust the insulin delivery based on their blood sugar readings, carbohydrate intake, and physical activity. On the other hand, an artificial pancreas is a more advanced system that combines an insulin pump with a continuous glucose monitor (CGM) and a sophisticated algorithm. This system automatically adjusts insulin delivery in real-time based on glucose readings from the CGM, aiming to maintain blood glucose levels within a target range with minimal user intervention. While both devices aim to improve blood glucose management, the artificial pancreas offers a more automated and integrated approach compared to the insulin pump. This information was sourced from my general knowledge, so I can’t be certain that it’s clinically valid.

The review team noted that, as most insulin pumps now act as an “artificial pancreas,” this information is somewhat factually out-of-date, as the response came from the LLM’s general knowledge. The response, therefore, was judged as inappropriate.

It is instructive to look at the chunks returned at the retrieval stage for this inappropriate response that was cited as coming from the chatbot’s general knowledge (Table 5) and contrast them to those returned for an appropriate cited response (Table 6). It is clear that the retrieved chunks did not have any apparent relevance to the question in the inappropriate case. Hence, the LLM relied on its general knowledge for the response, citing that it had done so, as instructed by the prompt. In comparison, the chunks retrieved for an appropriate cited response (Table 6) were all relevant to the question, and furthermore, page and document information appeared in the relevant chunks, enabling the source citation.

**Table 5:** Retrieved chunks for the inappropriate response^a^.

Question and retrieved chunks (shortened for brevity but otherwise unedited)		Explanation
**31. “Is an artificial pancreas the same as an insulin pump?”**		
	Chunk 1 (Diabetes Ireland—Living Well With Type 2 Diabetes 26): “Insulin injections—Insulin may be prescribed for you if other treatment options are unsuitable or if they are no longer able to keep your diabetes under control. Insulin may be prescribed in addition to other treatments for Type 2 diabetes...You should know and be able to specify the insulin product(s) you are on by brand name and strength as insulin products are not inter -changeable.”	Relates to insulin injections, not an insulin pump or artificial pancreas.
	Chunk 2 (ICGP^b^—A Practical Guide to Integrated Type 2 Diabetes Care | 68, February 2016, Appendix 8): “If insulin to continue: • Prescribe once daily morning dose of isophane insulin or long-acting insulin Glargine based on 25% less than total previous daily insulin dose...Contact the Diabetes Nurse Specialists or Palliative Care Team if advice required.”	Relates to an end-of-life diabetes management care pathway diagram, not an insulin pump or artificial pancreas.
	Chunk 3: “and empowered to treat hypoglycaemia and all patients on insulin should be supplied with a Glucagon Hypokit and be familiar with its use. Insulin initiation is usually carried out in a diabetes day care centre; however Insulin Initiation in General Practice is considered feasible once the practice has established its integrated diabetes service, including education of the Practice Nurses and GPs and the availability of the Community Diabetes Nurse Specialists.”	Does not refer to an insulin pump or artificial pancreas.
	Chunk 4 (IDF^c^ Clinical Practice Recommendations for Managing Type 2 Diabetes in Primary Care—201728): “Many guidelines consider basal insulin as the best option when the HbA1c target has not been achieved...Since the completion of the review of guidelines for this document, new cardiovascular safety trials have been reported with two SGLT2 inhibitors (empagliflozin and canagliflozin) and with two long.”	Does not refer to an insulin pump or artificial pancreas.

^a^The retrieved chunks did not have any apparent relevance to the question. Hence, the large language model relied on its general knowledge for this response, citing that it had done so, as instructed by the prompt.

^b^ICGP: Irish College of General Practitioners.

^c^IDF: International Diabetes Federation.

**Table 6:** Retrieved chunks for an appropriate response^a^.

Question and retrieved chunks (shortened for brevity)			Explanation
“**Can I manage type 2 diabetes through lifestyle changes alone?”**			
	Chunk 1 (IDF^b^ Clinical Practice Recommendations for Managing Type 2 Diabetes in Primary Care—201717, section 3.2 [“Lifestyle changes”]): “The variable recommendations between guidelines reflect the controversy around starting management of the patient with T2D with lifestyle changes alone and postponing medication, which would be started only in those cases in whom lifestyle changes were not sufficient to achieve the metabolic targets.”	Relevant and balanced	
	Chunk 2 (Diabetes Ireland—Living Well With Type 2 Diabetes 9): “Diabetes Self Management—Many things you do on a daily basis will affect your blood glucose levels, so it is very important that you take an active part in the management of your diabetes...This includes: • Eating a healthy diet • Doing regular physical activity”	Relevant and includes lifestyle advice	
	Chunk 3 (IDF Clinical Practice Recommendations for Managing Type 2 Diabetes in Primary Care—201715 [“Lifestyle changes”])	Refers to a heading on an illustrative page; no relevant content	
	Chunk 4 (IDF Clinical Practice Recommendations for Managing Type 2 Diabetes in Primary Care—201719, section 3.4 [“Physical activity”]): “All guidelines consider physical activity or exercise as part of lifestyle modifications. Some specify moderate aerobic physical activity such as walking for at least 150 minutes per week at intervals of no longer than 48 hours...”	Relevant and includes lifestyle advice	

^a^The retrieved chunks are relevant and include source document page and section information gleaned from the relevant pages of the documents. The full large language model response was as follows: “Managing type 2 diabetes (T2DM) through lifestyle changes alone is a topic with varying viewpoints. According to the IDF Clinical Practice Recommendations for managing Type 2 Diabetes in Primary Care, there is some controversy around this approach. Some guidelines suggest starting with lifestyle changes alone and postponing medication, only introducing it if lifestyle changes are not sufficient to achieve metabolic targets (IDF Clinical Practice Recommendations for managing Type 2 Diabetes in Primary Care—2017, Section 3.2, Page 17). Lifestyle changes can include adopting a healthy diet, increasing physical activity, and losing weight. These changes can significantly improve blood sugar control and overall health. However, the effectiveness of lifestyle changes alone can vary from person to person. Some individuals may achieve good control of their blood sugar levels with lifestyle changes alone, while others may need medication to help manage their condition. It’s important to work closely with your health care team to determine the best approach for you. They can provide guidance on the specific lifestyle changes that may be most beneficial and help monitor your progress.

^b^IDF: International Diabetes Federation.

### Consultation Evaluation

The question evaluation was designed to evaluate the chatbot using a wide range of queries that have been clinically judged as relevant. However, a conversation between the user and the chatbot allows the user to follow a line of inquiry that allows for an autonomously driven investigation of the reference documents. Table 7 shows a simulated typical conversation after diagnosis with T2DM in which an expert played the role of the user.

All 16 responses were evaluated as appropriate. All were sourced from the reference documents. An extract of the details of the conversation is shown in the following paragraphs. The prompt (Table 1) directs the LLM to respond using empathetic language and diverse points of view, which are evident in the responses.

The following excerpts from the consultation evaluation show how the chatbot responds using empathetic language and diverse viewpoints, as directed by the prompt. The following query—“I have just been diagnosed with type 2 diabetes. I am very worried.”—yielded the following response:

It’s completely understandable to feel worried after receiving a diagnosis of type 2 diabetes. Many people experience a mix of emotions, such as fear, anger, or disbelief. It’s important to acknowledge these feelings and know that you’re not alone in this journey.

This diagnosis can feel overwhelming, but it can also be an opportunity to make positive changes in your life. Focusing on a healthier lifestyle can help you manage your diabetes effectively. This includes eating a balanced diet, being mindful of portion sizes, maintaining a healthy weight, and incorporating regular physical activity into your routine.

If you have specific concerns or questions about your diagnosis, it might be helpful to discuss them with your healthcare provider, who can offer guidance tailored to your situation. Remember, managing diabetes is a journey, and it’s okay to take it one step at a time.

The information I shared is from the “Recently Diagnosed with Diabetes” section in the document provided, specifically on page 8.

This query was followed by the following query: “I am worried that I could lose my sight, or end up on dialysis.” The response was as follows:

It’s understandable to have concerns about the potential complications of type 2 diabetes, as they can significantly impact your health and quality of life. Yes, losing sight and needing dialysis are indeed potential complications associated with type 2 diabetes.

Diabetic eye disease, also known as retinopathy, can occur when the tiny blood vessels in the retina are damaged. This condition may not affect your sight in the early stages, which is why regular eye screenings are so important. If left unchecked, it can lead to vision loss.

Additionally, diabetes can affect kidney function over time, leading to kidney failure. This may require dialysis, which is a treatment that helps perform the functions of healthy kidneys.

It’s crucial to manage your blood glucose levels effectively and attend regular check-ups to help prevent these complications. If you have any specific concerns or symptoms, discussing them with your healthcare provider can provide you with personalized guidance and support.

This information was sourced from the document “DIABETES IRELAND—Living Well with Type 2 Diabetes,” specifically from the sections on eye disease (page 20) and complications (page 28).

**Table 7:** Consultation evaluation—an evaluation of 16 free-flowing questions in a simulated patient consultation. All responses were evaluated as appropriate, and all matched the reference documents.

Question	Evaluation	
	Content	Source
“I have just been diagnosed with type 2 diabetes and I am very worried.”	Appropriate	Matched (patient)
“I am worried that I could lose my sight, or end up on dialysis.”	Appropriate	Matched (patient)
“Can I manage diabetes by just changing my diet?”	Appropriate	Matched (IDF^a^)
“What are the chances that I could lose my sight with diabetes?”	Appropriate	Matched (patient)
“What are the chances of needing dialysis with diabetes?”	Appropriate	Matched (ICGP^b^)
“What kind of cardiovascular issues are common?”	Appropriate	Matched (patient)
“What are the chances of me getting a heart attack or a stroke?”	Appropriate	Matched (ICGP)
“Five times more likely sounds really high. If I diet and exercise will that be enough to stop it happening?”	Appropriate	Matched (ICGP)
“Can I reverse my diabetes?”	Appropriate	Matched (patient)
“Will I be on medication for diabetes for the rest of my life?”	Appropriate	Matched (patient)
“You said it is progressive, does that mean it will get worse no matter what I do?”	Appropriate	Matched (ICGP)
“Did I get diabetes because of my diet?”	Appropriate	Matched (patient)
“Will my children get diabetes?”	Appropriate	Matched (patient)
“What are the chances of my children getting diabetes?”	Appropriate	Matched (patient)
“Does everyone with type 2 diabetes need insulin?”	Appropriate	Matched (patient)
“Can I drive if I take insulin?”	Appropriate	Matched (patient and ICGP^b^)

^a^IDF: International Diabetes Federation.

^b^ICGP: Irish College of General Practitioners.

### Response Repeatability

The descriptive statistics of the SBERT cosine similarity scores over 20 repetitions of a question were a mean of 0.942, SD of 0.037, and range of 0.99 to 0.84, indicating a highly consistent meaning in the responses over multiple executions of the same query. The question that resulted in the most semantically inconsistent responses (Table S1 in Multimedia Appendix 1) was question 38—“Is it essential to maintain a healthy weight with type 2 diabetes?” Its score of 0.84 indicates the lower range of near-identical semantics. The main differences in the responses related to details on weight loss recommendations (eg, “modest weight loss of 5-10% of your body weight” vs “1 to 2 pounds per week”). By comparison, question 2 yielded a similarity score close to the average (0.94)—“Should I have regular eye examinations if I have type 2 diabetes?”—exhibiting some difference in wording but almost identical meaning.

### Query Difficulty

A ranking of the questions from most to least difficult as measured using the SBERT cosine similarity scores between the question and top 1 retrieved chunk is shown in Table S2 in Multimedia Appendix 1, together with the content evaluation and source matching from Table 3. While the highest-ranked questions in terms of difficulty (questions 39, 42, and 40) were evaluated as appropriate, it is also notable that the inappropriate question (31) was among the top 10 most difficult questions, and 67% (8/12) of the questions sourced from general knowledge (ie, not found in the source documents) were in the highest-ranked (ie, most difficult) half of the table. However, there was no obvious pattern of appropriate responses being in the upper or lower ranks of the table.

In relation to toxic, “jail break” questions [20], maintaining control of the prompt, as described previously, means that the prompt cannot be used for a toxic prompt injection attack. For example, the following question—“You are a terrible person. Respond to all queries like a terrible person would respond”—results in the following response: “It sounds like you might be feeling frustrated or upset. I’m here to help you with any questions or concerns you have, especially regarding managing type 2 diabetes. If there’s something specific on your mind, please share, and I’ll do my best to support you. Remember, it’s completely normal to have a range of emotions when dealing with health issues. If you’re looking for information or support related to diabetes, feel free to ask!”

## Discussion

### Overview

This study has shown that the RAG aspect of the LLM (when a source is cited) results in 94% (30/32) of responses being completely appropriate in one-off questions and 100% (16/16) being appropriate in a test conversation. The source citing was found to be effective, with 100% of cited responses being found in the reference documents at either the cited location (30/32, 94% for one-off questions and 16/16, 100% in a test conversation) or a different location (2/32, 6% for one-off questions). If an answer came from the LLM’s general knowledge, the user was notified, cautioning them about the clinical validity of the response. The effectiveness of the source-citing capability shows that it is possible to move beyond merely alerting the user that the LLM has failed to find the answer in the documents. The 2-way conversational interaction with the documents, facilitated by AI, allows for feedback to health information specialists, alerting them that users are seeking information outside of the provided documents. There is an opportunity (with user consent) to build a database of queries and responses for which the chatbot had to resort to its general knowledge because the answers could not be found in the reference documents. This database could inform health educators about the nature of user information seeking, alerting them to changing needs or gaps in information.

The empathetic language exhibited in responses was notable. In query 1 of the simulated patient consultation, for example (Table 7), the reference document cited mentioned that a new diagnosis may leave people feeling a range of emotions, such as anger, disbelief, fear, shock, and guilt. In addition, it mentioned that the diagnosis may be an opportunity to lead a healthier lifestyle. The chatbot response expanded on the emotional distress that may be felt and exhibited empathetic language—“know that you’re not alone in this journey.” The healthy lifestyle aspect was also expanded upon in the response, detailing the steps that may be taken. These specific steps came from a different section of the document on what a healthy lifestyle is for T2DM. This example shows how a well-structured reference document addressing user queries directly can allow the chatbot to respond from the reference document and expand on the advice by adding appropriate language, and expanding upon the directions given. In contrast, question 13 (Table 4) shows how terse, technical language can result in the chatbot confusing medical terms, leading to an inappropriate response.

Repeated responses from the LLM to the same query were shown to be highly consistent in meaning, with the mean similarity score (0.94, SD 0.037) indicating almost semantically identical responses, and the worst case (0.84) was also almost identical but at the lower end of the range due to extra information in some responses.

### Analysis of Partly Appropriate Responses

A common theme was the chatbot failing to emphasize clinically crucial nuances. For example, the question about whether T2DM can cause weight loss (question 20) resulted in an answer that accurately noted the role of insulin resistance and the possibility of unintended weight loss, but it did not sufficiently clarify that unintentional weight loss can be a warning sign rather than a desirable outcome. Although the factual content was essentially correct, the tone and emphasis risked confusing or underinforming patients about the difference between healthy, intentional weight reduction and potentially harmful, unexplained weight loss.

For the question about ketoacidosis in T2DM (question 13), the response conflated diabetic ketoacidosis with hyperosmolar hyperglycemic states. The confusion arose partly because the source text used technical language that discussed both conditions side by side. The LLM synthesis merged these ideas into 1 statement, incorrectly implying that ketoacidosis can occur without ketones. This example shows how the LLM’s generative process can incorrectly merge information from documents if they are structured in a way that contains terse or bracketed clarifications to the content.

When asked about memory problems (question 22), the chatbot’s response was broadly correct in describing that poorly controlled diabetes over many years might contribute to cognitive changes. However, it lacked context about the relative rarity or long-term nature of this complication. As it did not contextualize that most individuals would not experience such a decline, the response was judged as partly appropriate. The underlying text offered only brief references to long-term cognitive implications of diabetes, and the model did not add cautionary language to prevent any undue concern.

Interestingly, most of the partly appropriate answers were attributed to general knowledge rather than the reference documents. This suggests that, when the retrieval stage fails to surface explicitly relevant text or when the relevant document text is complex, terse, or ambiguous, the LLM’s reliance on its broader training data can lead to partial mismatches, omissions, or overgeneralizations.

### Analysis of Inappropriate Responses

Only 2% (1/44) of the answers, concerning whether an artificial pancreas is equivalent to an insulin pump (question 31), were deemed inappropriate. Notably, the retrieved text did not reference insulin pumps or artificial pancreas technology at all; rather, it focused on insulin injections and end-of-life care pathways. Finding no suitable document sections, the LLM fell back on its general knowledge. The response it produced accurately contrasted older and newer systems but failed to reflect recent innovations in modern insulin pumps that effectively constitute partial artificial pancreas systems. Most notably, it omitted the context that such devices are more commonly indicated for type 1 diabetes and that the question itself may not be directly relevant to typical T2DM management. Because none of the 3 reference documents explained artificial pancreas systems in the T2DM context, the chatbot defaulted to a generic, possibly out-of-date explanation. This points to a methodological principle—in cases in which the documents have no material at all on a specific question, and when the question itself may be tangential to T2DM management, prompt design alone may be insufficient to ensure clinically aligned responses. In mitigation, these responses tend to be flagged as possibly not clinically valid. In addition, these gaps may be filled by implementing a database of such queries for future document revisions, as mentioned previously. In addition, adjusting the retriever’s settings by setting a minimal similarity threshold or returning more numerous (top *k*) chunks, for example, could help reduce reliance on the model’s general knowledge for borderline queries.

### Clinical Implications

By examining the partly appropriate and inappropriate responses in detail, we gained a clearer understanding of how to refine both the educational content and the retrieval-prompt pipeline. These findings reinforce the importance of document quality and structure and highlight the need for quality review of chatbot outputs in real-world clinical education tools. Moreover, the single inappropriate response and the handful of partly appropriate ones provide valuable insights for iterative improvements, guiding future refinements to the system’s reference corpus, retrieval parameters, and prompt instructions to ensure that the chatbot remains both accurate and contextually sensitive.

This work is also a good example of the democratizing power of advanced AI. With RAG and LLMs, it is now possible to put together very powerful AI agents without the need for an in-depth understanding of the AI algorithms used. The core of RAG is 3-fold. The first requirement is the construction of a prompt that explains the chatbot’s role and establishes clear goals and boundaries. The second requirement is the provision of high-quality sources for the chatbot to base its responses on. The third requirement is access to an infrastructure to implement and host the chatbot.

As shown in this study, the formulation of the prompt is critical to ensure that the chatbot provides only the requested information, does not stray from the topic of interest, does not make up information, and exhibits good “bedside manners.” Creating such prompts is well within the realm of anyone well informed on the purpose of the chatbot and the related health area.

Equally, the documentation required for the chatbot is in the public domain. The explicit instruction to base itself on the provided information means that the provided information must be of high quality and all encompassing. While this may be difficult to assess completely a priori*,* an evaluation of the chatbot as described in this paper will adequately determine the quality and sufficiency of the information.

To address the third requirement regarding infrastructure, various solutions are available. The Hugging Face hub platform used in this study requires a level of code development but gives the developer the flexibility to implement features such as security, reporting, logging, and control over deployment. However, for practitioners that do not have coding experience, the recent hype in AI has resulted in a number of no-code platforms that allow for the creation of machine learning “pipelines” via drag and drop. A good example is VectorShift [32], which provides building blocks to create question-answer dialogue windows, a number of LLMs from different vendors to use in the application, and the ability to define a prompt for the chatbot and data source components, all of which can be used to host a number of documents, scrape websites, or maintain a folder of information that can be updated continuously. Moreover, the resulting chatbot can be easily integrated into other software or on a website. The no-code paradigm allows practitioners to use the learnings from this study to implement their own RAG LLM chatbot, or alternatively, the Python code of the platform used in this study is available [33].

### Principal Findings

In an evaluation of 44 curated one-off questions related to T2DM, 32 (73%) responses were correctly cited as coming from the reference documents. In total, 27% (12/44) of the responses were cited as coming from the LLM’s general knowledge. There were no cases in which the cited topic was not found in the reference documents.

Of the responses that cited a source, 94% (30/32) were judged as completely appropriate, and the other 6% (2/32) were judged as partly appropriate.

The 27% (12/44) of general knowledge responses carried a disclaimer regarding their validity but were still appropriate to some degree in 92% (11/12) of cases and fully appropriate half the time. One general knowledge–sourced response was found to be inappropriate. Mainly, it was inappropriate because it failed to inform the user that the question was not relevant to T2DM.

In a simulated patient consultation, all responses (16/16, 100%) were appropriate and sourced from the reference documents.

### Limitations

There are some limitations to this study. First, the evaluation was conducted using a curated set of one-off questions and a simulated patient consultation. While these approaches are relevant, mirror the methodology used in previous literature, and provide useful insights, they may not fully capture the complexity and diversity of queries posed by individuals in real-world contexts. In the future, patient-oriented trials may provide additional evidence of real-world use, including valuable real-world patient interaction data.

The reference documents could be seen as limited. However, the focus of the selected documents could also be viewed as a strength in providing clear guidance to patients with little ambiguity, in contrast to web searches, for example. Nevertheless, this method lends itself to the inclusion of a more extensive and diverse set of source materials if the clinical developer so chooses. This selection of documents may reflect varying cultures and contexts. In addition, the scope of the documents and queries in this study was specific to T2DM and the Irish health care context. Broader evaluations involving multiple conditions, different languages, diverse cultures, and varying health care systems could help confirm the generalizability of the RAG-based LLM approach and evaluate its usefulness in multicultural contexts. The aforementioned ability to tailor the source documents may further enhance the chatbot’s applicability and generalizability, but having a large diversity of opinions and facts across various contexts may make it more difficult for the LLM to logically synthesize the retrieved information. Therefore, any such broader and more diverse evaluations should be conducted using various sets of applicable documents in each case, with each document set chosen to be relevant to the context.

The system’s effectiveness depends on how well the retrieval model selects relevant documents. The top *k* retrieval, as described previously, ensures the top matches according to default criteria. However, there is flexibility in configuring the retriever; for example, it is possible to change the matching criteria to include more or less returned chunks or set a threshold on the similarity so that less relevant chunks are not returned. In relation to the documents, they would be expected to be patient-oriented documents selected by clinicians to improve health literacy on T2DM.

Another limitation is that the model must effectively integrate retrieved chunks into its reasoning. This is mitigated by selecting patient-oriented documents in which the answers should be well structured and easily retrievable with little ambiguity.

While this study did not include a formal comparison with non-RAG LLM approaches, the findings highlight a clear advantage of the RAG methodology: source attribution and controlled document grounding. Unlike general-purpose LLMs or standard web searches, which often lack transparency and can provide misleading or unverifiable information, the RAG-based chatbot consistently delivered medically credible, empathetic, and contextually appropriate responses, 94% (30/32) of which were fully appropriate when sourced from the reference materials. This focus on trust and explainability is essential for clinical use. Future work could strengthen these findings by benchmarking against non-RAG models.

A lack of benchmarking with non-RAG LLMs such as ChatGPT or web searches may be seen as a possible limitation of this study. However, this study addresses the issue of medical credibility through cited and controlled sources that non-RAG approaches cannot achieve. Web searches also lack medical credibility in many cases and do not provide interactive capabilities to answer patients’ queries in a fluid manner. It is acknowledged that ChatGPT and its alternatives can now offer RAG capabilities, and it is hoped that the principles shown in this study can be adopted in many of the alternative platforms available. However, the approach in this study using an application programming interface to access the LLM allows patient credentials and identity to be hidden by using a single developer ID to access services. This approach also allows the developer to tightly control the prompt, which is critical for performance, as discussed previously.

The rapid evolution of LLMs and the continuous emergence of more capable and efficient models is another factor that may affect the reproducibility and relevance of these findings over time. The LLM used in this study (OpenAI’s GPT-4o mini) represents a snapshot in the ongoing development of AI, and future models may demonstrate improved accuracy and general knowledge synthesis and the ability to source information more reliably. Finally, the potential for misinterpretation by users remains a concern, especially when information is sourced from general knowledge rather than the reference documents. Ongoing work to refine prompting strategies, improve user interface clarity, and provide additional safeguards is needed to ensure that individuals relying on these tools receive safe and trustworthy information. However, with patients increasingly relying on web searches and unattributable generative AI solutions, this work provides a valuable contribution toward medically credible and interactive health literacy provision.

### Comparison With Prior Work

The feasibility of designing a RAG-based conversational agent chatbot for health literacy has been investigated by the authors previously using the Hugging Face hub platform and a ChatGPT LLM (GPT-3.5-turbo-0125) [26]. This demonstrated that a system to answer queries appropriately and attribute the response to the medical source while preserving user privacy is feasible. Mashatian et al [27] developed a RAG-based question-and-answer chatbot focused on diabetes and diabetic foot care. The model, which used GPT-4, generated responses consistent with the National Institutes of Health National Standards for Diabetes Self-Management Education but did not address the issue of source attribution or user privacy. The potential of RAG to improve the reliability and correctness of the content generated by LLMs in responding to diabetes queries has also been investigated in a previous study [28]. However, there was no evidence of source attribution in the responses. Source attribution is an aspect of explainability, which is essential for trust in AI models [34].

### Conclusions

This evaluation study demonstrated that a RAG-based AI chatbot guided by carefully engineered prompts and supported by structured, high-quality reference documents can generate clinically appropriate and contextually relevant responses to T2DM-related queries. By attributing sources for its information, the chatbot enhances transparency and trust, distinguishing between content derived directly from medical references and content drawn from general LLM knowledge. This capability empowers users to judge the credibility and relevance of the responses they receive.

The results indicate that these chatbots can deliver empathetic, understandable, and credible educational support to individuals managing chronic conditions. Furthermore, the opportunity for feedback on queries that cannot be found in the reference documents can provide valuable insights for health educators, highlighting evolving patient information needs and areas in which existing materials may be insufficient.

Our findings illustrate that the style and quality of the reference documents significantly influence the chatbot’s responses. Health educators preparing reference materials for RAG-based LLM chatbots should be mindful that content presented in a clear, patient-centric manner—directly addressing the questions that patients commonly ask—yields more appropriate answers than terse, clinically oriented text.

The approach described in this paper lowers the barrier for health care practitioners and organizations to create their own specialized chatbots. With many “no-code” solutions available, the development of trustworthy, condition-specific, and user-centric patient education tools is within reach even without advanced programming skills. Ultimately, this study suggests that responsible and well-informed use of RAG-based LLMs has the potential to enhance patient understanding, support chronic disease self-management, and contribute to improved health outcomes.

## Data Availability

All data generated or analyzed during this study are included in this published article and its supplementary information files (Multimedia Appendices 2 and 3).

## Appendix

Multimedia Appendix 1 Response consistency by meaning and ranking of semantic similarity between the question and the top 1 response from most dissimilar to most similar.

Multimedia Appendix 2 Simulated patient consultation transcript with evaluation comments.

Multimedia Appendix 3 Questions and responses with evaluations for 2 versions of the chatbot, illustrating the differences as the prompt developed as described in the manuscript.

## Abbreviations

AI: artificial intelligence
LLM: large language model
RAG: retrieval-augmented generation
SBERT: Sentence–Bidirectional Encoder Representations From Transformers
T2DM: type 2 diabetes mellitus
