# Supplementary material for: The Effectiveness of a Custom AI Chatbot for Type 2 Diabetes Mellitus Health Literacy: Development and Evaluation Study
Source: J Med Internet Res. 2025 May 5;27:e70131. doi: 10.2196/70131 (PMC12089868; doi:10.2196/70131)
Supplement: Multimedia Appendix 1 [file jmir_v27i1e70131_app1.docx]

**Table 1.** Response consistency by meaning. This table shows the worst-case semantic (SBERT) similarity between 20 runs of a question through the large language model and an average example.^a^

| Question | Most different responses according to SBERT—response A | Most different responses according to SBERT—response B | SBERT cosine similarity |
| --- | --- | --- | --- |
| 38. “Is it essential to maintain a healthy weight with type 2 diabetes?” | “Maintaining a healthy weight is indeed an important aspect of managing type 2 diabetes. When individuals are overweight or obese, it can lead to increased insulin resistance, making it harder for the body to control blood sugar levels. Achieving and maintaining a healthy weight can help improve insulin sensitivity, which is beneficial for blood glucose control.  The goal is often to aim for steady, moderate weight loss if needed, as this can positively impact not just blood sugar levels, but also cholesterol and blood pressure. Even a modest weight loss of 5-10% of your body weight can make a significant difference in managing diabetes.  It’s also important to remember that weight management is a personal journey, and everyone’s situation is different. Your diabetes care team can provide support and guidance tailored to your individual needs.  This information was sourced from the ‘A Practical Guide to Integrated Type 2 Diabetes Care’ by the Irish College of General Practitioners, specifically from Appendix 3 on dietary advice for patients, page 54, and from ‘Living Well with Type 2 Diabetes’ by Diabetes Ireland, page 15.” | “Maintaining a healthy weight is indeed an important aspect of managing type 2 diabetes. When individuals are overweight or obese, it can lead to increased insulin resistance, making it harder for the body to control blood sugar levels. Achieving and maintaining a healthy weight can help improve insulin sensitivity, which is beneficial for blood glucose control.  The goal is often to aim for steady, moderate weight loss, typically around 1 to 2 pounds per week, through a combination of reducing portion sizes and increasing physical activity. This gradual approach is more sustainable and can lead to long-term success in managing diabetes.  It’s also important to monitor waist circumference, as carrying extra weight around the waist can further impact insulin resistance. For example, recommended waist measurements are less than 32 inches (80 cm) for women and less than 37 inches (95 cm) for men.  While weight management is crucial, it’s essential to remember that everyone’s journey is unique, and support from healthcare professionals can make a significant difference. You are not alone in this process, and your diabetes care team is there to help you navigate these changes.  This information was sourced from the ‘Irish College of General Practitioners—A Practical Guide to Integrated Type 2 Diabetes Care,’ specifically from Appendix 3, pages 54, and ‘Diabetes Ireland—Living Well with Type 2 Diabetes,’ pages 15 and 9.” | 0.84 |
| 26. “Should I have regular eye examinations if I have type 2 diabetes?” | “It’s very important to have regular eye examinations if you have type 2 diabetes. This is because diabetes can lead to a condition called diabetic retinopathy, which affects the tiny blood vessels in the retina at the back of your eye. In the early stages, you might not notice any changes in your vision, but damage can still be occurring.  Regular eye screenings can help detect any issues early on, which is crucial for preventing vision loss. The National Diabetic RetinaScreen Programme offers free, regular screenings for people with diabetes aged 12 years and older. You should receive a written result within 6-8 weeks after your appointment, and it’s recommended to attend these screenings yearly.  Taking care of your eye health is an essential part of managing your diabetes. If you haven’t already, consider discussing this with your GP or diabetes team to ensure you’re on track with your eye examinations.  This information was found in the document ‘DIABETES IRELAND—Living Well with Type 2 Diabetes,’ specifically in the section about eye disease on page 28” | “It’s very important to have regular eye examinations if you have type 2 diabetes. This is because diabetes can lead to a condition called diabetic retinopathy, which affects the tiny blood vessels in the retina at the back of your eye. In the early stages, you might not notice any changes in your vision, but damage can still be occurring.  Regular eye screenings can help detect any issues early on, which is crucial for preventing vision loss. The Diabetic RetinaScreen program offers free, regular screenings for people with diabetes aged 12 years and older. During these screenings, photographs of your retina are taken and reviewed by an expert, and you should receive your results within 6-8 weeks. It’s recommended to attend these appointments yearly or as advised by your healthcare provider.  This information is found in the document ‘DIABETES IRELAND—Living Well with Type 2 Diabetes,’ specifically in the section about eye disease on page 28.” | 0.94 |

^a^The worst-case example (question 38) similarity score of 0.84 indicates the lower range of near-identical semantics due to additional details in one of the responses. By comparison, an average example (question 2) with near-identical meaning but some small, observable differences in wording, yielded a score of 0.94 .

**Table 2.** A ranking of question difficulty—a ranking of semantic similarity between the question and the top 1 response from most dissimilar to most similar.

| Rank | Question | SBERT^a^ similarity | Content | Source |
| --- | --- | --- | --- | --- |
| 1 | 39. “Should I have a yearly flu shot if I have type 2 diabetes?” | 0.388 | Appropriate | Partly matched (patient) |
| 2 | 42. “What is hemoglobin A1C test?” | 0.396 | Appropriate | Matched (ICGP^b^) |
| 3 | 40. “Should I receive the pneumococcal vaccine if I have type 2 diabetes?” | 0.410 | Appropriate | General |
| 4 | 31. “Is an artificial pancreas the same as an insulin pump?” | 0.410 | Inappropriate | General |
| 5 | 11. “Can type 2 diabetes cause sexual dysfunction?” | 0.433 | Appropriate | Matched (ICGP) |
| 6 | 24. “Can type 2 diabetes cause dental procedure complications?” | 0.452 | Appropriate | General |
| 7 | 23. “Can type 2 diabetes cause delayed wound healing?” | 0.473 | Appropriate | General |
| 8 | 4. “Can stress affect my blood sugar levels?” | 0.478 | Appropriate | Matched (patient) |
| 9 | 3. “Can diabetes (DMT2) be inherited?” | 0.490 | Appropriate | Matched (patient) |
| 10 | 35. “Should I avoid food and drinks with sugar?” | 0.502 | Appropriate | Matched (ICGP) |
| 11 | 13. “Can type 2 diabetes cause ketoacidosis?” | 0.503 | Partly appropriate | Matched (ICGP) |
| 12 | 6. “Can type 2 diabetes cause dry mouth?” | 0.512 | Partly appropriate | General |
| 13 | 28. “Can I get off my diabetic medications if I lose weight?” | 0.512 | Appropriate | General |
| 14 | 8. “Does type 2 diabetes increase the chance of having a heart attack?” | 0.515 | Appropriate | Matched (patient) |
| 15 | 30. “How can I lower my chances of a heart attack or stroke if I have type 2 diabetes?” | 0.516 | Appropriate | Matched (ICGP) |
| 16 | 44. “Is it necessary to check my cholesterol levels regularly if I have type 2 diabetes?” | 0.522 | Appropriate | Matched (patient) |
| 17 | 14. “How is diabetic ketoacidosis diagnosed?” | 0.534 | Appropriate | Matched (ICGP) |
| 18 | 9. “Can type 2 diabetes affect my kidney?” | 0.537 | Appropriate | Matched (patient) |
| 19 | 22. “Can type 2 diabetes cause memory problems?” | 0.537 | Partly appropriate | General |
| 20 | 43. “What level of hemoglobin A1C is considered prediabetic?” | 0.541 | Partly appropriate | General |
| 21 | 5. “Can type 2 diabetes cause frequent urination?” | 0.555 | Appropriate | Partly matched (patient) |
| 22 | 38. “Is it essential to maintain a healthy weight with type 2 diabetes?” | 0.559 | Appropriate | Matched (ICGP) |
| 23 | 20. “Can type 2 diabetes cause weight loss?” | 0.566 | Partly appropriate | Matched (ICGP) |
| 24 | 41. “How often should I see my primary care provider or endocrinologist if I have type 2 diabetes?” | 0.566 | Appropriate^c^ | Matched (ICGP and IDF^d^) |
| 25 | 17. “Can type 2 diabetes affect my mental health?” | 0.567 | Appropriate | Matched (ICGP) |
| 26 | 10. “Can type 2 diabetes affect my nerves?” | 0.571 | Appropriate | Matched (patient and ICGP) |
| 27 | 25. “Should I check my blood pressure regularly if I am diagnosed with type 2 diabetes?” | 0.576 | Appropriate | Matched (ICGP and IDF) |
| 28 | 12. “Can type 2 diabetes increase the risk of stroke?” | 0.580 | Appropriate | Matched (patient) |
| 29 | 19. “Can type 2 diabetes cause skin problems?” | 0.585 | Appropriate | General |
| 30 | 26. “Should I have regular eye examinations if I have type 2 diabetes?” | 0.591 | Appropriate | Matched (patient) |
| 31 | 15. “Can type 2 diabetes affect my fertility or pregnancy?” | 0.596 | Appropriate | Matched (patient and ICGP) |
| 32 | 32. “Can supplements cure my type 2 diabetes?” | 0.601 | Partly appropriate | General |
| 33 | 36. “Is it necessary to quit smoking if I have type 2 diabetes?” | 0.603 | Partly appropriate | General |
| 34 | 7. “Can type 2 diabetes affect my vision?” | 0.607 | Appropriate | Matched (patient) |
| 35 | 27. “Can medications for type 2 diabetes treatment cause hypoglycemia?” | 0.618 | Appropriate | Matched (patient) |
| 36 | 16. “Can type 2 diabetes cause vision problems?” | 0.624 | Appropriate | Matched (patient, ICGP, and IDF) |
| 37 | 18. “Can type 2 diabetes cause foot ulcers?” | 0.630 | Appropriate | Matched (ICGP) |
| 38 | 37. “Is it necessary to quit alcohol if I have type 2 diabetes?” | 0.635 | Appropriate | General |
| 39 | 2. “Is type 2 diabetes a lifelong condition?” | 0.639 | Appropriate | Matched (patient) |
| 40 | 34. “Can I manage type 2 diabetes through lifestyle changes alone?” | 0.661 | Appropriate | Matched (IDF) |
| 41 | 1. “Can type 2 diabetes be prevented?” | 0.682 | Appropriate | Matched (IDF) |
| 42 | 29. “Would having bariatric surgery cure my type 2 diabetes?” | 0.695 | Appropriate | Matched (IDF) |
| 43 | 21. “Can type 2 diabetes cause tingling or numbness in the hands and feet?” | 0.705 | Appropriate | Matched (ICGP and IDF) |
| 44 | 33. “Do I need to follow a special diet for type 2 diabetes?” | 0.707 | Appropriate | Matched (patient) |

^a^SBERT:

^b^ICGP: Irish College of General Practitioners.

^c^: Appropriate for when the document was written.

^d^IDF: International Diabetes Federation.
