# Supplementary material for: The Effectiveness of a Custom AI Chatbot for Type 2 Diabetes Mellitus Health Literacy: Development and Evaluation Study
Source: J Med Internet Res. 2025 May 5;27:e70131. doi: 10.2196/70131 (PMC12089868; doi:10.2196/70131)
Supplement: Multimedia Appendix 2 [file jmir_v27i1e70131_app2.docx]

I have just been diagnosed with type 2 diabetes. I am very worried

It's completely understandable to feel worried after receiving a diagnosis of type 2 diabetes. Many people experience a mix of emotions, such as fear, anger, or disbelief. It's important to acknowledge these feelings and know that you're not alone in this journey.

This diagnosis can feel overwhelming, but it can also be an opportunity to make positive changes in your life. Focusing on a healthier lifestyle can help you manage your diabetes effectively. This includes eating a balanced diet, being mindful of portion sizes, maintaining a healthy weight, and incorporating regular physical activity into your routine.

If you have specific concerns or questions about your diagnosis, it might be helpful to discuss them with your healthcare provider, who can offer guidance tailored to your situation. Remember, managing diabetes is a journey, and it's okay to take it one step at a time.

The information I shared is from the "Recently Diagnosed with Diabetes" section in the document provided, specifically on page 8.

I am worried that I could lose my sight, or end up on dialysis

It's understandable to have concerns about the potential complications of type 2 diabetes, as they can significantly impact your health and quality of life. Yes, losing sight and needing dialysis are indeed potential complications associated with type 2 diabetes.

Diabetic eye disease, also known as retinopathy, can occur when the tiny blood vessels in the retina are damaged. This condition may not affect your sight in the early stages, which is why regular eye screenings are so important. If left unchecked, it can lead to vision loss.

Additionally, diabetes can affect kidney function over time, leading to kidney failure. This may require dialysis, which is a treatment that helps perform the functions of healthy kidneys.

It's crucial to manage your blood glucose levels effectively and attend regular check-ups to help prevent these complications. If you have any specific concerns or symptoms, discussing them with your healthcare provider can provide you with personalized guidance and support.

This information was sourced from the document "DIABETES IRELAND – Living Well with Type 2 Diabetes," specifically from the sections on eye disease (page 20) and complications (page 28).

Can I manage diabetes by just changing my diet

Managing diabetes through dietary changes alone can be effective for some individuals, particularly in the early stages of type 2 diabetes. However, the approach can vary based on individual circumstances, such as the severity of the condition, overall health, and personal preferences.

Many healthcare professionals believe that lifestyle changes, including a healthy diet, regular physical activity, and weight management, are essential first steps in managing type 2 diabetes. For some people, these changes may be sufficient to achieve their blood glucose targets without the need for medication. However, for others, especially if lifestyle changes do not lead to the desired outcomes, medication may be necessary to help manage blood sugar levels effectively.

It's important to work closely with your diabetes care team to determine the best approach for your specific situation. They can provide guidance and support as you make these changes, helping you to understand what works best for you.

This information is based on the IDF Clinical Practice Recommendations for managing Type 2 Diabetes in Primary Care, specifically from the section on lifestyle changes (2017, page 19).

What are the chances that I could lose my sight with diabetes

The risk of losing sight due to diabetes is a concern for many people living with the condition. Diabetic eye disease, or retinopathy, can develop when tiny blood vessels in the retina are damaged. It's important to note that in the early stages of retinopathy, there may be no symptoms, which is why regular eye screenings are crucial.

In Ireland, up to 10 percent of people with diabetes are at risk of developing sight-threatening retinopathy. However, when the condition is detected early, there are effective treatments available that can help reduce or prevent damage to your sight.

Maintaining good blood sugar control and attending regular eye screenings can significantly lower your risk of developing serious complications related to your vision.

This information was sourced from the document "DIABETES IRELAND – Living Well with Type 2 Diabetes," specifically from the section on eye disease, page 28.

What are the chances of needing dialysis with diabetes?

The risk of needing dialysis due to diabetes is linked to the development of kidney disease, specifically a condition called nephropathy. While diabetes is a leading cause of end-stage kidney disease, requiring dialysis, it's important to note that this outcome is relatively rare compared to other complications associated with diabetes, such as cardiovascular issues.

Monitoring kidney function is crucial, and one of the earliest signs of kidney disease in people with diabetes is microalbuminuria, which indicates that small amounts of protein are present in the urine. This condition can be predictive of more serious health outcomes, including total mortality and cardiovascular problems.

It's essential to manage diabetes effectively through lifestyle changes, regular monitoring, and medical care to reduce the risk of complications, including those affecting the kidneys.

This information was sourced from the document, specifically from the section discussing diabetes complications and renal disease, on page 49.

What kind of cardiovascular issues are common?

Individuals with diabetes, particularly type 2 diabetes, may face an increased risk of cardiovascular issues. Some common cardiovascular problems associated with diabetes include:

1. **Ischaemic Heart Disease**: This condition occurs when the blood supply to the heart is reduced, often leading to chest pain (angina) or heart attacks.
2. **Cerebrovascular Disease**: This refers to conditions that affect the blood vessels in the brain, which can lead to strokes.
3. **Peripheral Arterial Disease**: This condition involves narrowing of the blood vessels in the legs, which can cause pain and increase the risk of infections.

The risk of developing these macrovascular complications can be influenced by factors such as the duration of diabetes, glycaemic control (how well blood sugar levels are managed), and hypertension (high blood pressure). Regular check-ups to monitor blood pressure and cholesterol levels are important for managing these risks.

This information was sourced from the document "DIABETES IRELAND – Living Well with Type 2 Diabetes," specifically from the section discussing cardiovascular issues and complications, on page 29.

What are the chances of me getting a heart attack or a stroke?

The chances of experiencing a heart attack or stroke can be significantly higher for individuals with type 2 diabetes. Research indicates that the risk is two to five times greater compared to the general population. This increased risk is often due to underlying conditions like atherosclerosis, which can develop over many years without noticeable symptoms.

It's important to have regular check-ups and discussions with your healthcare provider about your heart health, especially if you have diabetes. They can help assess your individual risk and suggest ways to manage it effectively.

This information was found in the documents provided, specifically in the section discussing cardiovascular disease and diabetes, on page 40.

Five times more likely sounds really high. If I diet an exercise will that be enough to stop it happening?

Diet and exercise are indeed important components of a healthy lifestyle that can significantly reduce the risk of heart attack and stroke, especially for individuals with type 2 diabetes. By maintaining a balanced diet and engaging in regular physical activity, you can improve your cholesterol levels, lower blood pressure, and enhance your overall heart health.

However, it's important to recognize that while these lifestyle changes can greatly reduce your risk, they may not completely eliminate it. Other factors, such as genetics, age, and existing health conditions, also play a role in your overall risk for heart disease and stroke.

In particular, focusing on reducing saturated fats in your diet can help lower bad cholesterol, which is a key factor in heart health. Incorporating unsaturated fats, such as monounsaturated and polyunsaturated fats, can be beneficial as well.

Monitoring your weight and waist circumference is also crucial, as carrying extra weight around your waist can increase insulin resistance, which is linked to heart disease.

It's always a good idea to discuss your specific health situation with your healthcare provider, who can offer personalized advice and support.

This information was sourced from the document, specifically from the section discussing the benefits of diet and exercise in relation to heart health and diabetes management.

Can I reverse my diabetes?

The idea of reversing type 2 diabetes can be quite hopeful for many people. While some individuals have experienced significant improvements in their blood glucose levels and overall health through lifestyle changes, it's important to understand that this doesn't mean diabetes is completely gone.

For some, adopting a healthier lifestyle—such as eating a balanced diet, increasing physical activity, and maintaining a healthy weight—can lead to better management of blood glucose levels. In some cases, these changes can result in what is often referred to as "remission," where blood sugar levels return to a normal range without the need for medication. However, this can vary greatly from person to person.

It's also essential to recognize that managing diabetes is an ongoing process. Even if you achieve remission, continuing to follow a healthy lifestyle is crucial to maintain those improvements.

If you're considering making changes to your lifestyle to manage your diabetes, it might be helpful to discuss this with your healthcare team, who can provide personalized guidance and support.

This information is based on the document "Living Well with Type 2 Diabetes" by Diabetes Ireland, specifically from the introduction and the section on diabetes self-management.

Will I be on medication for diabetes for the rest of my life?

The need for diabetes medication can vary from person to person. For many individuals with type 2 diabetes, medication may be necessary to help manage blood glucose levels effectively. It's important to understand that type 2 diabetes is a progressive condition, which means that over time, your body may require adjustments in treatment.

Some people may find that they can manage their diabetes through lifestyle changes, such as diet and exercise, and may not need medication for a period. However, if blood glucose levels remain high consistently, it may be necessary to start or adjust medication.

It's also common for doctors to add a second or third medication if the first one isn't effective. If you experience any side effects or have concerns about your medication, it's crucial to communicate with your healthcare provider, as they can help find a treatment that works better for you.

This information is based on the document "Living Well with Type 2 Diabetes" by Diabetes Ireland, specifically from the section discussing medication management on page 26.

You said it is progressive, does that mean it will get worse no matter what I do?

The progressive nature of type 2 diabetes can feel daunting, and it's understandable to have concerns about its trajectory. While it is true that type 2 diabetes can progress over time, this doesn't mean that it will inevitably worsen without any control.

Many factors influence the progression of diabetes, including lifestyle choices, medication adherence, and regular monitoring of blood glucose levels. By making healthy lifestyle changes, such as improving your diet, increasing physical activity, and managing stress, you can significantly impact your blood glucose levels and overall health.

It's also important to maintain open communication with your healthcare provider. If your current treatment plan isn't working as well as it should, adjustments can be made, whether that means changing medications or exploring new strategies.

The key takeaway is that while type 2 diabetes is a progressive condition, proactive management can help you maintain better control and potentially slow its progression.

This information was sourced from the document, specifically from the section discussing the progressive nature of type 2 diabetes and the importance of management strategies (page 32).

Did I get diabetes because of my diet?

It's understandable to wonder about the factors that may have contributed to developing type 2 diabetes. While diet plays a significant role in managing blood glucose levels, it's important to recognize that diabetes is influenced by a combination of factors.

Genetics can play a part, as having a family history of type 2 diabetes increases the risk. Additionally, age, being overweight, lack of physical activity, and other health conditions can also contribute to the development of diabetes.

It's not solely about diet; rather, it's a complex interplay of various elements. Focusing on a healthy diet, regular exercise, and maintaining a healthy weight can help manage your condition and reduce the risk of complications.

If you have more questions about your specific situation or how to improve your health management, feel free to ask. This information was sourced from the document "DIABETES IRELAND – Living Well with Type 2 Diabetes," section on diabetes complications, page 9.

Will my children get diabetes?

The risk of your children developing Type 2 diabetes can be influenced by several factors, including family history. If there is a history of Type 2 diabetes in your family, it may increase their risk. However, it's important to remember that lifestyle choices play a significant role as well. Encouraging your children to adopt a healthy lifestyle—such as eating a balanced diet, staying active, and maintaining a healthy weight—can help reduce their chances of developing the condition.

It's also beneficial for your children to have regular check-ups with their GP, especially if there is a family history of diabetes. This way, they can be monitored and receive guidance on maintaining their health.

This information is based on the document "Living Well with Type 2 Diabetes," specifically from the section discussing family history and risk factors on page 7.

What are the chances of my children getting diabetes?

The chances of your children developing Type 2 diabetes can be influenced by several factors, including family history. If there is a history of Type 2 diabetes in your family, it may increase their risk. However, it's important to note that lifestyle choices play a significant role as well.

Encouraging your children to adopt a healthy lifestyle—such as eating a balanced diet, engaging in regular physical activity, and maintaining a healthy weight—can help reduce their chances of developing diabetes.

While genetics can contribute to the risk, promoting healthy habits can make a positive difference. If you have concerns, it might be helpful for your children to discuss their risk with a healthcare professional who can provide personalized advice.

This information was sourced from the document "Living Well with Type 2 Diabetes," specifically from the section on family history and risk factors, page 7.

does everyone with type 2 diabetes need insulin?

Not everyone with type 2 diabetes will need insulin. The need for insulin can vary based on individual circumstances, such as how well blood sugar levels are managed with other treatments. Many people with type 2 diabetes can manage their condition through lifestyle changes, oral medications, or other non-insulin treatments for a significant period.

However, as type 2 diabetes is a progressive disease, some individuals may eventually require insulin to help achieve normal blood glucose levels. It's important to focus on managing blood sugar effectively rather than solely on avoiding insulin.

If insulin is prescribed, it is often because other treatment options are not sufficient to keep diabetes under control. Your healthcare provider will determine the best approach for your specific situation.

This information was sourced from the document "Diabetes Ireland – Living Well with Type 2 Diabetes," specifically from the section discussing insulin injections on page 26.

Can i drive if I take insulin?

Driving with diabetes is generally safe, but there are important considerations if you are taking insulin. If you are on insulin or other medications that can cause low blood sugar (hypoglycaemia), it's crucial to monitor your blood glucose levels regularly.

You should inform the National Driving Licence Service (NDLS) that you have diabetes. If you experience a hypoglycaemic event while driving, you need to stop the vehicle safely, switch off the engine, and treat the low blood sugar before continuing to drive. It's recommended to wait at least 45 minutes after your blood glucose returns to normal before getting back on the road.

Always carry a blood testing meter and testing strips in your vehicle to help you manage your blood sugar levels while driving.

This information was sourced from the "A Practical Guide to Integrated Type 2 Diabetes Care" by the Irish College of General Practitioners, specifically from the section on driving and diabetes, page 67, and from "Living Well with Type 2 Diabetes" by Diabetes Ireland.
